# Neuromuscular Junction Changes in a Mouse Model of Charcot-Marie-Tooth Disease Type 4C

**DOI:** 10.3390/ijms19124072

**Published:** 2018-12-17

**Authors:** Silvia Cipriani, Vietxuan Phan, Jean-Jacques Médard, Rita Horvath, Hanns Lochmüller, Roman Chrast, Andreas Roos, Sally Spendiff

**Affiliations:** 1 John Walton Muscular Dystrophy Research Centre, Newcastle University, Newcastle upon Tyne NE1 3BZ, UK; cipriani.silvia@hsr.it; 2 INSPE-Institute of Experimental Neurology, San Raffaele Scientific Institute, 20132 Milan, Italy; 3 Division of Neuroscience, San Raffaele Scientific Institute, 20132 Milan, Italy; 4 Leibniz-Institut für Analytische Wissenschaften -ISAS- e.V.; Otto-Hahn-Strasse 6b, 44227 Dortmund, Germany; vietxuan.phan@gmail.com (V.P.); andreas.roos@isas.de (A.R.); 5 Department of Neuroscience, Karolinska Institutet, 171 65 Stockholm, Sweden; jean-jacques.medard@ki.se (J.-J.M.); roman.chrast@ki.se (R.C.); 6 Department of Clinical Neuroscience, Karolinska Institutet, 171 65 Stockholm, Sweden; 7 Department of Clinical Neurosciences, University of Cambridge, John Van Geest Cambridge Centre for Brain Repair, Forvie, Robinson way, Cambridge Biomedical Campus, Cambridge CB2 0PY, UK; rh732@medschl.cam.ac.uk; 8 Department of Neuropediatrics and Muscle Disorders, Medical Center-University of Freiburg, Mathildenstrasse 1, 79106 Freiburg, Germany; hlochmuller@cheo.on.ca; 9 Centro Nacional de Análisis Genómico, Center for Genomic Regulation, Barcelona Institute of Science and Technology, Baldri I reixac 4, 08028 Barcelona, Spain; 10 Children’s Hospital of Eastern Ontario Research Institute, University of Ottawa, Ottawa, ON K1H 8L1, Canada; 11 Division of Neurology, Department of Medicine, The Ottawa Hospital, Riverside Drive, Ottawa, ON K1H 7X5, Canada; 12 Department of Neuropediatrics, Developmental Neurology and Social Pediatrics, Centre for Neuromuscular Disorders in Children, University Children’s Hospital Essen, University of Duisburg-Essen, 45122 Essen, Germany

**Keywords:** Charcot-Marie-Tooth disease 4C, SH3TC2, neuromuscular junction, mouse models, peripheral neuropathy, demyelination

## Abstract

The neuromuscular junction (NMJ) appears to be a site of pathology in a number of peripheral nerve diseases. Charcot-Marie-Tooth (CMT) 4C is an autosomal recessive, early onset, demyelinating neuropathy. Numerous mutations in the *SH3TC2* gene have been shown to underlie the condition often associated with scoliosis, foot deformities, and reduced nerve conduction velocities. Mice with exon 1 of the *Sh3tc2* gene knocked out demonstrate many of the features seen in patients. To determine if NMJ pathology is contributory to the pathomechanisms of CMT4C we examined NMJs in the gastrocnemius muscle of SH3TC2-deficient mice. In addition, we performed proteomic assessment of the sciatic nerve to identify protein factors contributing to the NMJ alterations and the survival of demyelinated axons. Morphological and gene expression analysis of NMJs revealed a lack of continuity between the pre- and post-synaptic apparatus, increases in post-synaptic fragmentation and dispersal, and an increase in expression of the gamma subunit of the acetylcholine receptor. There were no changes in axonal width or the number of axonal inputs to the NMJ. Proteome investigations of the sciatic nerve revealed altered expression of extracellular matrix proteins important for NMJ integrity. Together these observations suggest that CMT4C pathology includes a compromised NMJ even in the absence of changes to the innervating axon.

## 1. Introduction

Charcot-Marie Tooth (CMT) disease was first reported over a hundred years ago [1]. Research in a well-defined Norwegian population demonstrated a prevalence of 1/2500 [2] suggesting it is one of the most frequent forms of inherited neurological disorders [3]. Several genetic defects have been linked to the demyelinating forms of the disease [3,4,5,6,7], which can be dominant (CMT1) or recessive (CMT4). CMT subtype 4C (CMT4C) is an autosomal recessive form of demyelinating neuropathy usually with an early onset [3,7,8]. The clinical hallmarks comprise distal muscular atrophy, scoliosis, distal weakness, foot deformities (pes cavus), and reduced motor and sensory nerve conduction velocities [3,4,5,9,10]. However, the age of onset and disease severity may change within and between families, thus making genotype–phenotype correlations difficult. The disease locus for CMT4C is on chromosome 5q23-33 [4,5], and both nonsense and missense mutations in the *SH3TC2*/*KIAA1985* gene have been shown to be causative for CMT4C [3,4,6]. Over 70 causative mutations for CMT4C have been described with the R954X mutation shown to be particularly common [3,7]. Expression of SH3TC2 has recently been described in in cultured Schwann cells [11]. It is a well conserved 144 kDa protein containing Scr homology 3 (SH3) and tetratricopeptide repeat (TRP) domains, suggesting a role as scaffold protein [6,12]. Its role in myelination is supported by its localisation to the plasma membrane and to the perinuclear endocytic recycling compartment [8,12,13,14].

The Sh3tc2 knock out mouse (*Sh3tc2^ΔEx1/ΔEx1^*) [8] was created to better understand the pathophysiology of CMT4C. These mice show development, life-span, and fertility similar to those of wild type and heterozygous littermates and while they do not develop scoliosis, they do harbour peripheral nervous system abnormalities. At 4 weeks of age the homozygous animals’ manifest typical features of neuropathy such as reduction of motor nerve conduction velocity and sensory nerve conduction velocity, thus suggesting a possible impairment of fiber myelination. At six months of age they have an abnormal clenching of toes and clasping of hindlimbs upon tail suspension. Electron (EM) and light microscopy of the sciatic nerve shows hypomyelination, changes at nodes of Ranvier, and in older mice occasional onion bulb formation and abnormal Schwann cell branches [8]. The resulting abnormal organization of the nodes of Ranvier found both in *Sh3tc2^ΔEx1/ΔEx1^* mice and in CMT4C patients, seems to support the role of SH3TC2 in myelination.

Notably, it has been shown that SH3TC2 interacts with the guanosine triphosphatase Rab11, which is known to be involved in the recycling of internalised membranes and receptors back to the cell surface [8,13]. In this scenario, SH3TC2 acts as a Rab11 effector molecule, suggesting that *SH3TC2* mutations lead to a disruption of this interaction resulting in myelination impairment [13,14]. In the peripheral nervous system, the Neuregulin-1/ErbB signalling pathway is important for myelination during early post-natal development. *SH3TC2* acts as a regulator of ErbB2 receptor internalisation that is required for myelination and mutations in this gene have been demonstrated to prevent its promotion of internalisation [15]. Taken together, the evidence suggest an important role of endosomal recycling process in myelin sheath formation as myelination requires dramatic changes in the cellular architecture of differentiating glia and a high degree of cell polarization [16].

Morphological findings obtained from EM analysis done on human sural nerve biopsies from patients with CMT4C, show non-myelinating Schwann cell complexes with abnormal cell processes, demyelinated-remyelinated axons surrounded by onion bulbs made up of empty basal lamina sheaths, and Remak bundles [3,5,10].

There is currently no treatment for CMT4C patients and existing approaches rely on trying to reduce symptoms through orthopaedic surgery, special shoes for ankle support, and physical exercise [3,17]. Affected individuals are overseen by several different specialists including neurologists, physiotherapists and orthopaedists [17].

Paramount to finding potential therapies for CMT4C is the understanding of the exact mechanisms of pathogenesis of the disease.

The neuromuscular junction (NMJ) has recently been shown to be dysfunctional in many conditions which do not necessarily have the NMJ as a primary site of pathogenesis [18,19,20]. NMJ dysfunction, structural changes, and delayed maturation have also been observed in animal models of CMT1A [21,22], and CMT2D [23,24]. Treatments for some causes of NMJ dysfunction are available [25], and thus the NMJ could represent a therapeutic target for some of these conditions, as has been shown in a mouse model of CMT2D [24]. In a number of the above conditions Schwann cells, specifically Terminal Schwann cells (tSC) at the NMJ, have been hypothesised to contribute to the observed pathology [26]. Given the expression of SH3TC2 in Schwann cells, and the importance of tSC for NMJ maintenance and recovery, we hypothesised that NMJ dysfunction could contribute to the pathology observed in CMT4C. We examined this possibility using the *Sh3tc2*^Δ*Ex1*/Δ*Ex1*^ mouse model, which was previously shown to be a useful in vivo model to study the disease mechanisms of CMT4C [8].

## 2. Results

**Figure 1:**
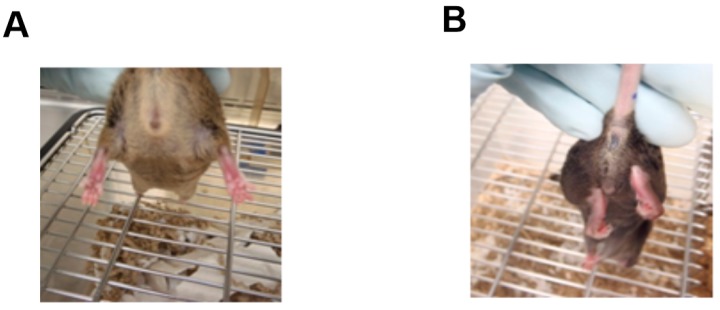
Hindlimb abnormalities in *Sh3tc2*^Δ*Ex1*/Δ*Ex1*^ mice. Suspension of mice by their tails shows normal positioning of the limbs and toes in control animals (**A**) and abnormal clenching of the toes in *Sh3tc2*^Δ*Ex1*/Δ*Ex1*^ mice (**B**).

To determine if NMJ dysfunction may be a significant contributing factor to the pathomechanisms of CMT4C we examined tissue taken from *Sh3tc2*^Δ*Ex1*/Δ*Ex1*^ mice and compared it to that from control animals (heterozygous *Sh3tc2*^Δ*Ex1/+*^ mice). This included structural and transcriptional analysis of the NMJ and proteomic investigations of the sciatic nerve. The *Sh3tc2*^Δ*Ex1/+*^ mice have been previously described [8,15] and show a mild phenotype, which includes normal development, lifespan, and fertility. However, on suspension they demonstrate an abnormal clenching of the hindlimbs (Figure 1) and they also have reduced nerve conduction velocities [8].

### 2.1. NMJ Structural Changes in Sh3tc2^Δ^^Ex1^^/Δ^^Ex1^ Mice

NMJ labelling was performed in gastrocnemius muscle from eight-month healthy control and affected *Sh3tc2*^Δ*Ex1*/Δ*Ex1*^ mice. The labelling was performed on whole mount muscle using neurofilament to label the nerve, synaptophysin to indicate the pre-synaptic terminal and α-bungarotoxin to identify the post-synaptic endplate. Analysis was performed blinded to the animal group and used a standardised methodology, “NMJ-morph”, which consists of a series of morphometric analysis comprising measurements of the nerve, pre-/post-synapses and their structural alignment [27]. In addition to the variables calculated using NMJ-morph, we also recorded the number of NMJs in which no pre-synaptic structure (synaptophysin or neurofilament staining) was observed, despite a post-synaptic endplate (α-bungarotoxin) being visible, these are sometimes referred to as “vacant” or “denervated” NMJs [19,21]. The following data is presented as mean ± SEM with data for control animals being given first.

**Figure 2:**
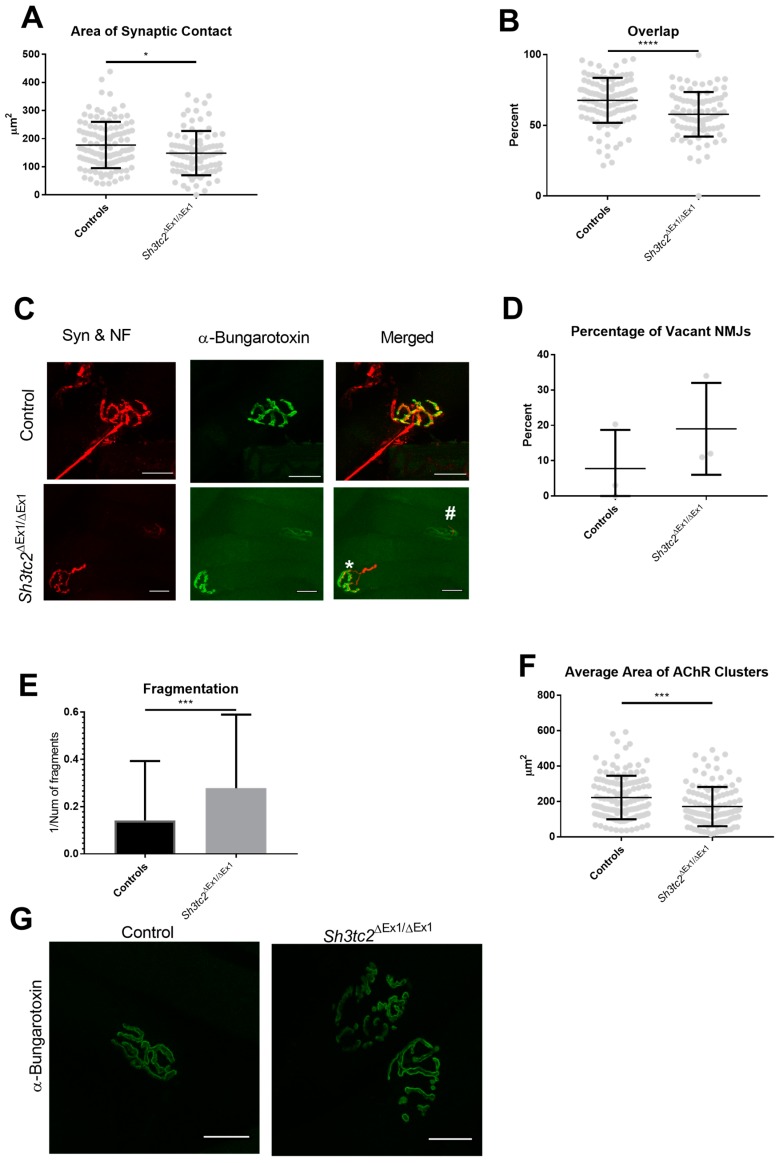
Changes in neuromuscular junction (NMJ) “alignment” and fragmentation at the endplate of *Sh3tc2*^Δ*Ex1*/Δ*Ex1*^ (*n* = 3) mice compared to control (*n* = 3) animals. (**A**) The area of synaptic contact between the pre- and post-synaptic apparatus is reduced in the NMJs of *Sh3tc2*^Δ*Ex1*/Δ*Ex1*^ mice (NMJs *n* = 91) when compared to control mice (NMJs *n* = 115) (Mann-Whitney test); (**B**) In addition, the percentage overlap, defined as the extent to which the nerve terminal overlaps the AChRs, was reduced in *Sh3tc2*^Δ*Ex1*/Δ*Ex1*^ mice (NMJs *n* = 115) when compared to control animals (NMJs *n* = 91) (Mann-Whitney test); (**C**) Reduced overlap in *Sh3tc2*^Δ*Ex1*/Δ*Ex1*^ animals. Control images show an NMJ with 75% overlap, *Sh3tc2*^Δ*Ex1*/Δ*Ex1*^ images show 50% (*) and 28% (#) overlap. NF = Neurofilament, Syn = synaptophysin; (**D**) The number of “vacant” NMJs, defined as an endplate with no pre-synaptic staining present, was not increased in the *Sh3tc2*^Δ*Ex1*/Δ*Ex1*^ animals (*n* = 3) compared to control animals (*n* = 3) (unpaired *t*-test); (**E**) The fragmentation index, the number of discrete AChR clusters present at the endplate, was increased in *Sh3tc2*^Δ*Ex1*/Δ*Ex1*^ (NMJs *n* = 114) vs. control (NMJs *n* = 132) animals (Mann-Whitney test); (**F**) There was a concomitant decrease in average size of AChR clusters in *Sh3tc2*^Δ*Ex1*/Δ*Ex1*^ (NMJs *n* = 114) vs. control (NMJs *n* = 133) NMJs (Mann-Whitney test). (**G**) Images showing increased fragmentation, reduced average size of AChR clusters, and some dispersal of the endplate. Graphs show mean ± SEM, scale bar is 25 μm, * *p* < 0.05, *** *p* < 0.0005, **** *p* < 0.0001.

Analysis of the degree of overlap revealed a lack of alignment between the pre- and post-synaptic apparatus in *Sh3tc2*^Δ*Ex1*/Δ*Ex1*^ animals. With *Sh3tc2*^Δ*Ex1*/Δ*Ex1*^ mice having a significantly smaller area of synaptic contact (control 177.4 ± 7.665 μm^2^ vs. *Sh3tc2*^Δ*Ex1*/Δ*Ex1*^ 148.4 ± 8.248 μm^2^ (mean ± SEM)) (Figure 2A). This resulted in a significantly reduced “overlap” percentage in the *Sh3tc2*^Δ*Ex1*/Δ*Ex*^ mice (67.69 ± 1.481% vs. 57.81 ± 1.649%) (Figure 2B,C). While there was an increase in the percentage of NMJs that were “vacant” (absent neurofilament and synaptophysin staining), in the *Sh3tc2*^Δ*Ex1*/Δ*Ex1*^ mice, this was not significant (Figure 2D). There was an increase in the degree of fragmentation in *Sh3tc2*^Δ*Ex1*/Δ*Ex*^ mice (0.1415 ± 0.0219 vs. 0.2784 ± 0.02916) (Figure 2E), determined by dividing 1 by the number of fragments; the increase in fragmentation also contributed to the significant reduction in the average area of acetylcholine receptor (AChR) clusters (222.4 ± 10.67 μm^2^ vs. 171.4 ± 10.42 μm^2^) (Figure 2F,G).

**Figure 3:**
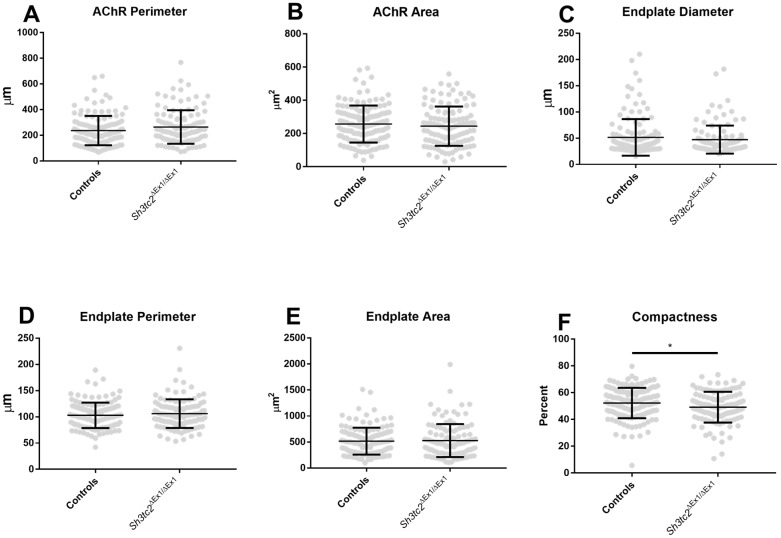
Post-synaptic changes at the endplate of *Sh3tc2*^Δ*Ex1*/Δ*Ex1*^ (*n* = 3) versus control (*n* = 3) mice: there were no differences between *Sh3tc2*^Δ*Ex1*/Δ*Ex1*^ (NMJ *n* = 114) and control mice (NMJ *n* = 133) in terms of (**A**) AChR perimeter (Mann-Whitney test); (**B**) AChR area (Mann-Whitney test); (**C**) Endplate diameter (Mann-Whitney test); (**D**) Endplate perimeter (Mann-Whitney test) and; (**E**) Endplate area (Mann-Whitney test); (**F**) There was a slight but significant reduction in the compactness of the NMJs in *Sh3tc2*^Δ*Ex1*/Δ*Ex1*^ animals (unpaired *t*-test). Graphs show mean ± SEM, * *p* < 0.05.

Following the observation of changes in alignment of the pre- and post-synaptic apparatus, we examined pre- and post-synaptic structures in more detail. There was a slight but non-significant increase in the AChR perimeter of *Sh3tc2*^Δ*Ex1*/Δ*Ex1*^ animals (Figure 3A) which could be attributed to the increase in fragmentation seen above. There were no differences between control and *Sh3tc2*^Δ*Ex1*/Δ*Ex1*^ animals in terms of AChR area (Figure 3B), or the endplate region, which encompasses all the AChRs and the space between those receptors (Figure 3B–E). However, the compactness of the endplate, determined by dividing the AChR area by the endplate area was found to be significantly reduced in the *Sh3tc2*^Δ*Ex1*/Δ*Ex1*^ animals (Figure 3E,F) (52.18 ± 0.9829% vs. 49.15 ± 1.077%), suggesting there was dispersal of the endplate (Figure 2G). 

**Figure 4:**
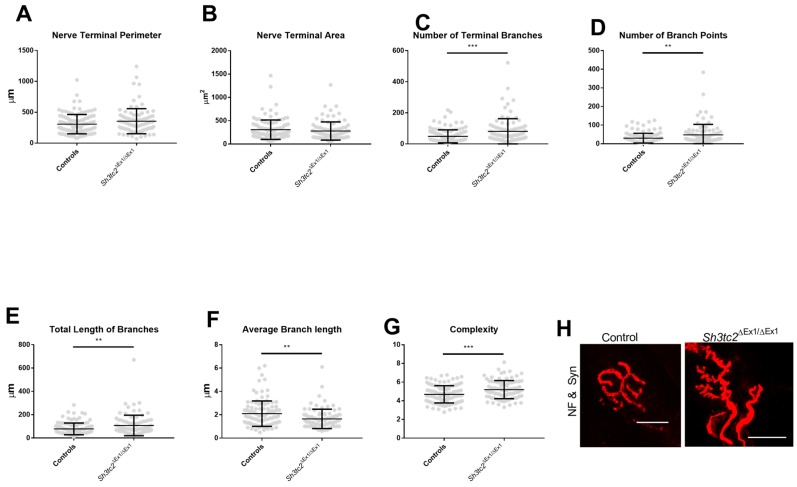
Pre-synaptic changes at the NMJs of *Sh3tc2*^Δ*Ex1*/Δ*Ex1*^ (*n* = 3) compared to control (*n* = 3) mice. There were no changes in the *Sh3tc2*^Δ*Ex1*/Δ*Ex1*^ animals (NMJ *n* = 91) compared to control (NMJ *n* = 118) in terms of: (**A**) Nerve terminal perimeter (Mann-Whitney test) or; (**B**) Nerve terminal area (Mann-Whitney test); (**C**) The number of terminal branches was increased in *Sh3tc2*^Δ*Ex1*/Δ*Ex1*^ animals (NMJ *n* = 91) compared to control (NMJ *n* = 117) mice (Mann-Whitney test); (**D**) This is also reflected in the increase in branching points in *Sh3tc2*^Δ*Ex1*/Δ*Ex1*^ animals compared to control mice (Mann-Whitney test); (**E**) The total length of all branches was increased in the *Sh3tc2*^Δ*Ex1*/Δ*Ex1*^ animals compared to control animals while the average length of these branches was reduced (Mann-Whitney test) (**F**). The complexity of the NMJs was increased in *Sh3tc2*^Δ*Ex1*/Δ*Ex1*^ animals (Mann-Whitney test) (**G**). (**H**) In the *Sh3tc2*^Δ*Ex1*/Δ*Ex1*^ animals, many small projections can be seen, but the length of these projections is not long (NF = Neurofilament & Syn = Synaptophysin). Graphs show mean ± SEM, scale bar is 25 μm, ** *p* <0.005, *** *p* < 0.0005.

In terms of pre-synaptic changes there were no significant differences in nerve terminal perimeter or area between control and *Sh3tc2*^Δ*Ex1*/Δ*Ex1*^ mice (Figure 4A,B). However, there was a significant increase in the number of terminal branches (48.67 ± 3.884 vs. 80.9 ± 8.513) and therefore not surprisingly branch points (29.78 ± 2.436 vs. 47.62 ± 5.922) (Figure 4C,D). While this increase in branching gave a significant increase in total length of branches (78.61 ± 4.641 μm vs. 107.4 ± 9.222 μm) (Figure 4E), the average length of these branches was significantly reduced (2.094.4 ± 0.1009 μm vs. 1.645 ± 0.08686 μm) (Figure 4F). The increase in branching of the *Sh3tc2*^Δ*Ex1*/Δ*Ex1*^ mice as seen in the increase in branching and branchpoints contributed to a significant increase (4.688 ± 0.08565 vs. 5.189 ± 0.1016) in the complexity of their NMJs (Figure 4G,H).

**Figure 5:**
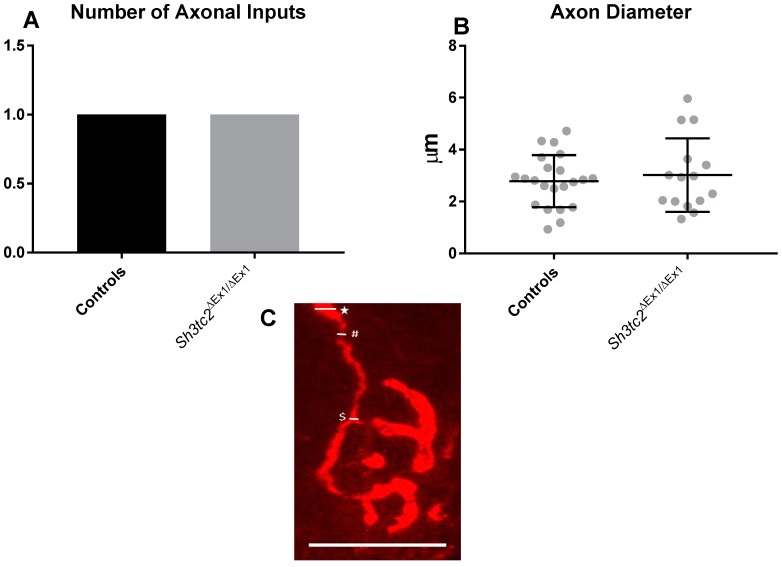
Axonal parameters in *Sh3tc2*^Δ*Ex1*/Δ*Ex1*^ (*n* = 3) and control (*n* = 3) animals: (**A**) Only single axonal inputs were observed for both *Sh3tc2*^Δ*Ex1*/Δ*Ex1*^ (NMJs *n*= 15) and control (*n* = 22) animals, therefore no SEM is given; (**B**) There were no differences in the axon diameter (unpaired *t*-test). (**C**) Axon inputs were identified with neurofilament staining and the diameter determined by measuring the axon at its widest (*) and thinnest (#) points, and where the axon ramifies ($). Graphs are mean ± SEM, scale bar is 25 μm.

Interestingly there was an absence of axonal changes, with the number of axonal inputs and axon width not being significantly different between control and *Sh3tc2*^Δ*Ex1*/Δ*Ex1*^ mice (Figure 5A–C).

The above analysis revealed a reduction in the compactness of the post-synaptic endplate, increased post-synaptic fragmentation, increased branching of the pre-synaptic terminal, and a reduction in the degree of overlap of the pre- and post-synaptic apparatus. Taken together this demonstrates a clear alteration of the NMJ structure independent of axonal changes (no reduction in axon width). To explain these structural changes at the NMJ of *Sh3tc2*^Δ*Ex1*/Δ*Ex1*^ mice, we examined RNA transcripts of genes of interest in NMJ plasticity. Whereas skeletal muscle of 8 months old animals was investigated to identify perturbations of NMJ-architecture, qPCR-studies were performed on muscle samples derived from 6 months old animals. This approach aimed to identify factors altered at an earlier time-point that we hypothesize to contribute to the NMJ structural changes.

### 2.2. Gene Expression Analysis of Denervation and Reinnervation Markers

**Figure 6:**
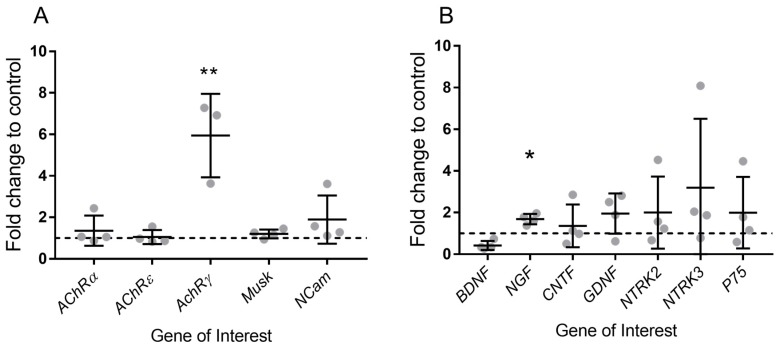
Denervation and reinnervation markers in *Sh3tc2*^Δ*Ex1*/Δ*Ex1*^ (*n* = 4) and control (*n* = 4) mice. QPCR analysis of; (**A**) Denervation markers: *AChRα, AChRε, AChRγ, MuSK and NCam* and of reinnervation markers: *BDNF, NGF, CNTF, GDNF, NTRK2, NTRK3, p75* in gastrocnemius muscle of 6 months old control and *Sh3tc2*^Δ*Ex1*/Δ*Ex1*^ mice (unpaired *t*-test) (**B**). Values for *Sh3tc2*^Δ*Ex1*/Δ*Ex1*^ animals were normalized to control animals, dashed line = 1 (control animals). Graphs are mean ± SEM, * *p* < 0.05, ** *p* < 0.005.

Gene expression analysis of denervation and reinnervation markers, performed by qPCR on gastrocnemius muscle from *Sh3tc2*^Δ*Ex1*/Δ*Ex1*^ mice, revealed the presence of significant variations. The *AChRγ* subunit, often increased in adult cases of denervation, was significantly increased (Figure 6A) in *Sh3tc2*^Δ*Ex1*/Δ*Ex1*^ mice (1.003 ± 0.1887 vs. 5.943 ± 1.161). The *Sh3tc2*^Δ*Ex1*/Δ*Ex1*^ mice group had one result removed as an outlier (determined using the ROUT method, GraphPad Prism), however, this value was a 236-fold increase on the control animals and thus its removal does not alter the pattern of increased *AChRγ* seen in the *Sh3tc2*^Δ*Ex1*/Δ*Ex1*^ mice. The remaining denervation markers (*AChRα*, *AChRε*, *Muscle-Specific Kinase* (*MuSK*)), and *Neural cell adhesion molecule* (*NCam*)) did not show any significant variations. In terms of markers of NMJ reinnervation, *Nerve growth factor* (*NGF*) was significantly upregulated (1 ± 0.1436 vs. 1.685 ± 0.1243) in *Sh3tc2*^Δ*Ex1*/Δ*Ex1*^ mice. While several other gene transcripts were increased, there was considerable variation in the samples, and no significant differences were seen in *brain-derived neurotrophic factor* (*BDNF*), *cilliary neurotrophic factor* (*CNTF*), *glial cell derived neurotrophic factor* (*GDNF*), *neurotrophic receptor tyrosine kinase 2* and *3* (*NTRK2* & *3*), and *p75* (Figure 6B*)*. 

While morphological analysis revealed structural changes at the NMJ, the above results also indicated there is a certain amount of denervation occurring in the animals, an interesting finding given the absence of axonal changes. However, the denervation may be compensated by the increased expression of *NGF* as an attempt to restore a functional innervation. Accordingly, the molecular findings appear to support the morphological alterations seen through the confocal microscopy analysis of the NMJs. To further explain the changes we saw at the NMJ and in particular the absence of changes in the axon width and number of axonal inputs, we investigated the proteomic signature of sciatic nerves derived from eight-month-old *Sh3tc2*^Δ*Ex1*/Δ*Ex1*^ animals, filtering for proteins relevant to NMJ-function and integrity.

### 2.3. Relevant Findings in the Proteomic Signature of SH3TC2-deficient Nerves from Sh3tc2^Δ*Ex1*/Δ*Ex1*^ Animals

We expected the pre-synaptic NMJ structural changes we observed in Sh3tc2^ΔEx1/ΔEx1^ animals to be accompanied by axonal changes that contributed to this disruption, as previously reported [21,23,24]. However, the number of axonal inputs and axonal width were unchanged in *Sh3tc2^ΔEx1/ΔEx^*^1^ animals, furthermore, pre-synaptic changes that increased the complexity of the terminal occurred. While transcriptional analysis suggested that these observations could be attributed to an increase in neurotrophins identified by the study of gastrocnemius muscle, the NMJ is a tightly controlled functional unit that is responsive to signaling from both pre and post-synaptic tissues. We therefore wanted to examine both the possible mediators of the observed NMJ alterations earlier in the motor control pathway and proteins preventing the preservation of axon diameter upon demyelination. Applied proteomics represents a very powerful tool to obtain unbiased insights into the pathophysiology of diseases such as neuromuscular disorders [28]. Here, we made use of this technique to gain further molecular insights into NMJ-pathology as well as prevention of axonal degeneration. Doing so, our filtered data revealed changes of 20 relevant proteins out of a total of 40 altered in abundance (50%) in the sciatic nerves of *Sh3tc2^ΔEx1/ΔEx1^* mice. These proteins were selected according to their known functions and thus feasible impact in axonal preservation as well as NMJ-vulnerability and are presented in Table 1. All information about these proteins was extracted from uniprot (www.uniprot.org, accessed on 9 October 2018). Interestingly most of the proteins that were elevated in the *Sh3tc2*^Δ*Ex1*/Δ*Ex1*^ animals are associated with the extra-cellular matrix (ECM).

## 3. Discussion

CMT4C is a demyelinating form of inherited neuropathy, with patients suffering from a combination of symptoms including muscular atrophy, distal weakness, foot deformities, scoliosis, and reduced motor and sensory nerve conduction velocities [3,4,9]. Its prevalence is estimated at 0.7/100,000 making it the most common type of recessive CMT [7]. The lack of treatment for CMT4C suggests that more information on the pathomechanisms of the disease are needed. The objective of our study therefore was to determine if NMJ changes are present in a mouse model of CMT4C and consequently could present a novel treatment target for the disease.

The list of neuromuscular diseases that are known to have some involvement of the NMJ, either as a primary or secondary site of pathogenesis is growing [18,19,21,22,23,24,29]. While amyotrophic lateral sclerosis (ALS) is predominantly characterised by the loss of upper and lower motor neurons, the SOD1^G93A^ mouse has demonstrated integrity of the NMJ is important in the pathomechanisms of the condition with NMJ alterations appearing early in the disease course [19]. These abnormal alterations include a decrease in NMJ complexity, an increase in fragmentation, and a decrease in the overlap of pre-and post-synaptic apparatus, in addition to axonal changes. While remaining innervated, NMJs from mice models of spinal bulbar muscular atrophy are fragmented, have a wider synaptic cleft, and abnormal synaptophysin distribution [18]. A reduction in NMJ density, degree of overlap, AChR cluster size, and increases in fragmentation have also been reported in mouse models of CMT1A and 2D [21,22,23,24]. Our phenomenological analysis demonstrating an increase in endplate fragmentation and dispersal, increased pre-synaptic branching, and a reduction in the degree of overlap of pre- and post-synaptic apparatus, suggest CMT4C should now be added to the list of neuropathies that are accompanied by NMJ alterations. Given the Schwann cell pathology demonstrated in *Sh3tc2*^Δ*Ex1*/Δ*Ex1*^ mice [8,15] and the importance of tSC for the NMJ this is not unexpected. Future studies should investigate this hypothesis through the examination of tSC at the NMJ in these animals to provide more information on the mechanisms causing these changes. This could be performed through EM, immunolabeling of tSCs and the NMJ, or even through proteomic investigations following laser dissection of NMJs from tissue. While the NMJ is a tightly controlled unit, with specialised pre- and post-synaptic regions required to operate in a coordinated manner, it is pharmacologically accessible and responsive. Promising results from animal models of neuropathy suggest a concerted effort to improve NMJ function may alleviate some patient’s symptoms’ [29,30].

NMJ analysis performed on gastrocnemius muscle from eight months-old *Sh3tc2*^Δ*Ex1*/Δ*Ex1*^ mice, revealed interesting and significant changes at the NMJs. The analysis followed a standardised morphometric protocol (“NMJ-morph”), which evaluates several separate pre- and post-synaptic variables and derived measurements [27]. The automated and standardised nature of this approach allows for many NMJs to be investigated, therefore increasing the likelihood of uncovering differences between groups. In this case, some of the most striking observations were decreases in overlap of the pre- and post-synaptic apparatus, increases in fragmentation, and a reduction in NMJ compactness in *Sh3tc2*^Δ*Ex1*/Δ*Ex1*^ animals. We had a slight, though non-significant increase in “vacant” NMJs, which has been seen previously in mouse models of CMT [21,23]. Although unlike other models, in the current investigation axonal degradation was not observed [21,23,24]. Although it would be interesting to examine the axons of *Sh3tc2*^Δ*Ex1*/Δ*Ex1*^ animals using EM to see if ultrastructure alterations are present that are undetectable by confocal microscopy.

While many post-synaptic changes were observed in *Sh3tc2*^Δ*Ex1*/Δ*Ex1*^ animals, increases in post-synaptic dispersal and fragmentation, as previously seen in CMT mouse models [24], a number of changes were pre-synaptic. Specifically, in *Sh3tc2*^Δ*Ex1*/Δ*Ex1*^ animals we observed an increase in terminal branches, branch points, and total length of branches. However, this growth was accompanied by a reduction in the average length of these branches suggesting that these “new” branches remained short. The increased branching observed in *Sh3tc2*^Δ*Ex1*/Δ*Ex1*^ animals led to an increase in “complexity” of the pre-synaptic terminal, but this was not matched by the insertion of new AChRs as can be seen by the reduction in “overlap”. It is possible that the increase in branching and resultant complexity observed in the *Sh3tc2*^Δ*Ex1*/Δ*Ex1*^ animals was an attempt at adaptation of the NMJ to maintain/prevent the loss of neuromuscular transmission. While structural changes are not in themselves always indicative of a decrease in NMJ transmission [31], they have been linked to denervation and NMJ dysfunction [24].

Interestingly, previous research demonstrating NMJ structural abnormalities in CMT animal models has shown strong muscle location specificity of the NMJ loss, and the resulting muscle fiber atrophy and motor impairment [21,23]. The prospect that this was linked to myosin heavy chain (MHC) fiber types has been examined [22,32] but ultimately did not reveal anything. However, the possibility that different muscles are more or less susceptible to denervation due to their MHC fiber type proportions has not been extensively investigated. This question could also be examined in *Sh3tc2*^Δ*Ex1*/Δ*Ex1*^ animals though the study of muscles with differing MHC compositions, for example the soleus versus the extensor digitorium longus. The selective vulnerability of distal muscles to NMJ loss has also been proposed as a potential explanation behind the muscle susceptibility in these diseases [33]. The extent and timing of NMJ dysmorphology, has been shown to be specific to the gene disrupted in the animal model, with the severity of the model correlating with the degree of NMJ pathology [23,24]. It would be interesting to examine the changes we observed in the *Sh3tc2*^Δ*Ex1*/Δ*Ex1*^ mice in terms of distal verses proximal muscles, as well as the timings of these changes to determine if this dysmorphology is occurring early or late in the disease course or is even a developmental deficit.

To obtain molecular insights into the structural NMJ perturbations in SH3TC2-deficient mice, a transcript analysis of denervation and reinnervation markers was performed in gastrocnemius muscle using qPCR. The results obtained from the gene expression analysis of denervation (*AChRα, AChRε, AChRγ, MuSK and NCam*) and reinnervation (*BDNF, NGF, CNTF, GDNF, NTRK2, NTRK, p75*) markers supported the results of the structural analysis. An increase *AChRγ* has often been seen in cases of denervation and NMJ dysfunction, as well as CMT [2,23]. Although we did not observe changes in *CNTF, BDNF,* and *MuSK*, that have previously been observed in cases of surgical denervation and neurotrypsin overexpression [34,35], suggesting the extent and mode of denervation modulates the gene expression. While *AChRγ* was increased in *Sh3tc2*^Δ*Ex1*/ΔEx1^ animals, the absence of axonal changes, mild neuropathic phenotype (abnormal toe clenching and clasping of hindlimbs at 6 months), and lack of changes in many of the transcriptional markers of denervation, suggests that only a limited amount of denervation is occurring. We hypothesize that while this limited denervation may contribute to the alterations at the NMJ it cannot account for the full extent of NMJ changes, which may be mediated by alterations in the tSCs. Analysis of muscle indicators of denervation and reinnervation, for example fiber type grouping, grouped fiber atrophy, myosin heavy chain co-expressing fibers, or the presence of small angular fibers may give a more complete picture to the extent of denervation occurring in the *Sh3tc2*^Δ*Ex1*/Δ*Ex1*^ animals.

Transcript analysis also revealed the potent growth factor *NGF* was increased, potentially reflecting the increase in NMJ branching seen in *Sh3tc2*^Δ*Ex1*/Δ*Ex1*^ animals. The increased expression of *NGF* may be explained as an attempt to restore NMJ function, as neurotrophins are known to have important roles in neural development, maintenance, and maturation. Indeed, previous studies have demonstrated that they are present at the NMJ and play a central role in promoting motor axon sprouting and innervation in response to denervation, particularly *BDNF, NGF,* and *GDNF* [8,9,34,35]. As its name suggests, *NGF* is primarily involved in the growth, as well as the maintenance, proliferation, and survival of nerve cells, which undergo apoptosis in its absence [36]. It rapidly increases a few days after a lesion and remains elevated for up to 2 weeks [37] suggesting the increase we observed was a result of a recent or ongoing changes at the synapse. NGF stimulates cell survival through the activation of several intracellular pathways, in particular, it leads to the activation of the PI3K-Akt and MAPK pathways, which both result in transcriptional increased expression of anti-apoptotic proteins. Consequently, *NGF* may be a viable target for a therapeutic intervention.

Transcript analysis was performed in whole gastrocnemius muscle from the mice, while changes occurring at individual NMJ may be mediated locally from the underlying nucleus. It is possible that this approach masked some of the changes and thus follow up experiments could examine transcripts from laser dissected NMJs. If this was coupled with immunostaining, then correlations may also be found with structural changes.

The proteomic signature of SH3TC2-deficient sciatic nerves from *Sh3tc2*^Δ*Ex1*/Δ*Ex1*^ animals was studied to correlate our morphological findings, suggestive of NMJ-vulnerability in the absence of degenerating demyelinated axons, with changes in abundances of proteins. GFAP has been identified as a protein with increased abundance in the sciatic nerves of the CMT4C mouse model and increases of this protein has already been described in the aetiology of other neuropathies [38] and is important for nerve regeneration [39]. In addition, the increase of FIBB, FIBG and MFAP2 might indicate a compensatory mechanism towards prevention of axonal degeneration as fibrin gel conduits improve re-myelination, axon ingrowth and innervation in a nerve-regeneration model [40]. As OLFL3 is important for functional organization of the nervous system [41], its increase in SH3TC2-deficient nerves also accords with activation of neuronal pro-survival mechanisms. This concept is further supported by the observed increase of *NGF* transcript level (see above) concomitant with TGM2, two proteins working in concert in cytoprotection and neurite outgrowth [42], as well as by an increase of APOD [43], APOE [44], SYNPO [45], and RABP2 [46]. The possible protective role on the axon of these sciatic nerve proteins could be investigated further through their targeting in cell and animal models that demonstrate axonal degeneration in response to de-myelination.

Regarding the vulnerability of NMJs to the loss of SH3TC2, increases of five different collagens suggest the activation of molecular mechanisms to prevent the complete breakdown of the NMJ. These proteins have been linked to structural and functional integrity as well as regeneration of the NMJ [47,48]. Moreover, collagens promote acetylcholine receptor clustering [49] and transcripts encoding for the gamma-subunit of the AChR were increased in gastrocnemius muscle of diseased animals (see above). C1QB is also increased in sciatic nerves derived from *Sh3tc2*^Δ*Ex1*/Δ*Ex1*^ mice and complement activation at the motor end-plates in amyotrophic lateral sclerosis has been described as a disease modifier [50]. Since homozygous LAMA5 mutations have been linked to a pre-synaptic form of congenital myasthenic syndrome associated with myopia, facial tics, and failure of neuromuscular transmission [51], increase of this protein in SH3TC2-deficient nerves supports the concept of prevention of NMJ-breakdown in *Sh3tc2*^Δ*Ex1*/Δ*Ex1*^ animals. In the same context, MATN2 (also increased in SH3TC2-deficient nerves) modulates axon guidance [52] and may, therefore, contribute to the observed increase in nerve branches in gastrocnemius muscles of *Sh3tc2*^Δ*Ex1*/Δ*Ex1*^ animals [53]. The pathomorphological observation that these increased branches are shortened might correlate with the decrease of IF5A2 (www.uniprot.org/uniprot/Q8BGY2, accessed on 9 October 2018).

We have demonstrated at the structural, transcriptional, and protein level, that *Sh3tc2*^Δ*Ex1*/Δ*Ex1*^ mice undergo synaptic vulnerability that may contribute to the pathogenic mechanisms of the disease while axons seem to be preserved. Our data provide basis for the further characterization of NMJ changes in CMT4C patients and indicate that *Sh3tc2^ΔEx1/ΔEx1^* mice represent a relevant model to evaluate potential therapeutic approaches aiming at preserving the synapses between moto neuron axons and muscles. It would be interesting to further explore these NMJ changes by treatment of the mice with anticholinesterase drugs, to see if this modifies the NMJ alterations observed here. If performed in humans harboring the CMT4C mutations it could also ascertain if NMJ changes are present in patients and if they represent a viable therapeutic target.

## 4. Materials and Methods 

### 4.1. Materials Animals

Mice were housed with 12-h light/dark cycles and had access to water and standard chow ad libitum. Genotyping was performed as previously described [8]. All animal work was performed in accordance with the Swedish regulations and approved by the regional ethics review committee Stockholm, Stockholms norra djurförsöksetiska nämnd (number N79/15, approved 29 September 2015). Mice were sacrificed after anesthetic overexposure by cervical dislocation.

### 4.2. NMJ Structural Analysis Using Whole Mount Muscle 

NMJ staining was performed using gastrocnemius muscle from eight months-old *Sh3tc2^ΔEx1/ΔEx1^* mice according to standard procedures [23,27]. On the day of collection muscles were fixed overnight in 4% paraformaldehyde, then washed twice in phosphate buffered saline (PBS) and stored in PBS until labelling. To label the NMJs, muscles were first separated into small fiber bundles (5–10 fibers) under a stereomicroscope. Samples were permeabilized in ethanol and methanol for 10 min (−20 °C). Samples were incubated in blocking solution (5% horse serum, 5% Bovine Serum Albumin (BSA), 1% Triton X-100 in PBS) for 4 h at room temperature (RT) with gentle agitation. Muscles were incubated with primary antibodies overnight (4 °C) on a rocking table, all diluted in blocking solution without triton X-100 (Rb pAB to Synaptophysin (Abcam, Cambridge, UK, 1:100 dilution) and Mouse mAb to Neurofilament (Cell Signalling, Danvers, MA, USA, 1:100 dilution)). The following day, samples were incubated for a further 2 h (RT) and washed 4 × 1 h in blocking solution. Samples were then incubated overnight in the dark (4 °C) with secondary antibodies (Alexa Fluor 594 Goat anti-Rabbit Immunoglobin (IgG) (Thermo Fisher Scientific, Waltham, MA, USA, 1:200 dilution) and Alexa Fluor 594 Goat anti-mouse IgG1 (Thermo Fisher Scientific, 1:200 dilution)), together with Alexa Fluor 488-conjugated α-bungarotoxin (1:250). Finally, the muscles were washed 4 × 1 h in PBS and mounted on slides using Vectashield Hard Set™ medium for fluorescence (Vector Laboratories, Burlington, CA, USA) and covered with a cover slip. Imaging was performed using a Nikon eclipse-T Inverted Confocal Microscope (Nikon, Tokyo, Japan) (512 × 512 frame size, ×60 magnification, 2 μm z-stack interval, and sequential image acquisition). All NMJs visible in the sample were imaged, with an average of 42 ± 9 (mean ± SEM) NMJs being analysed per sample. Analysis was performed on maximum intensity projections according to a recently published protocol [27] using Image J/Fiji (ImageJ 1.51 K, Java 1.6.0_24, NIH, Bethesda, MD, USA) [33].

### 4.3. Gene Expression Studies

To try and determine the events preceding any changes in NMJ structure gene expression studies were performed on gastrocnemius muscle from 4 control and 4 *Sh3tc2^ΔEx1/ΔEx1^* mice at six months of age. 

### 4.4. RNA Extraction and Conversion

RNA isolation was performed using a standard phenol-chloroform protocol. The tissue was weighed and one volume of trizol was added and the samples homogenised on ice using a Tissue Ruptor (QIAGEN Instruments Venio, The Netherlands). Samples were incubated for 5 min (RT) and centrifuged (4 °C) for 10 min at 12,000× *g*. Next, 0.2 mL of chloroform/volume of trizol was added to the cleared homogenate solution and the sample vigorously shaken by hand for 15 s. Samples were then incubated for 2–3 min (RT) and centrifuged at 12,000× *g* for 15 min (4 °C). The aqueous phase containing the RNA was transferred into a fresh tube. Then 0.5 mL/volume of trizol of isopropyl alcohol was added to each sample which was shaken by hand and incubated for 10 min (RT). Samples were centrifuged at 12,000× *g* for 15 min (4 °C) to obtain a pellet containing RNA which was washed in 75% ethanol and centrifuged at 12,000× *g* for 5 min (4 °C). The pellet was dried in a heat block (55–60 °C), dissolved in RNase-free water and incubated for 10 min. Samples were assessed for purity and concentration using a Thermo Fisher Scientific Nanodrop 2000 and then underwent treatment for DNA removal using the Ambion Life tech DNA-Free Kit™ (Thermo Fisher Scientific, Waltham, MA, USA). Reverse Transcription was performed using the Applied Biosystems High Capacity cDNA Reverse Transcription kit (Thermo Fisher Scientific. A master mix composed of: 10× RT Buffer, 25× dNTPs Mix, 10× RT Random Primers, MultiScribe RT and Nuclease-free H_2_O was prepared. 10 µL of master mix was mixed with 10 µL of RNA. Samples were briefly centrifuged and placed onto a thermal cycler using the following protocol: 10 min at 25 °C, 120 min at 37.5 °C, 5 min at 85 °C, and at 4 °C until use. 

### 4.5. Real-Time PCR (qPCR)

To determine the optimum conditions for the qPCR reaction a “pooled” cDNA sample containing 2 µL of each sample was made and used to determine the primer annealing temperature, cDNA dilution, and efficiency of the reaction (Supplementary Table S1). Tata Box Binding protein was chosen as reference gene as it has been shown to be one of the most stable genes in myogenic cells [54,55,56]. The qPCR reaction was performed using Power SYBR^®^ Green PCR Master Mix (Thermo Fisher Scientific) with 2 µL of diluted cDNA and 18 µL of master mix composed of 6 µL of DEPC-H_2_O, 1 µL of forward primer, 1 µL of reverse primer (Supplementary Table S1) and 10 µL of SYBR Green. Samples and no-template control (NTC) were run in triplicate. Experiments were performed on a BIO-RAD CFX machine (Bio-Rad Laboratories, Berkeley, CA, USA) under the following conditions: 40 cycles (95 °C for 10 min, 95 °C for 15 s, primer annealing temperature for 30 s), with a melt curve performed at the end to check for contamination and primer dimer (65 °C to 95 °C with increment of 0.5 °C for 5 s). Samples were only included if the melt curve gave a single clear peak, the sample triplicates were consistent, and the cycle threshold CT values were at least 3 CT from the NTC. Data was analysed using the ΔΔCq method.

### 4.6. Sample Preparation for Proteomics

Nerve tissues derived from eight-month-old *Sh3tc2^ΔEx1/ΔEx1^* and control (heterozygous) mice were snap-frozen in liquid nitrogen, followed by homogenization in 0.3 mL lysis buffer composed of 50 mM Tris-HCl (pH 7.8), 150 mM NaCl and 1 % sodium dodecyl sulfate (SDS), supplemented with complete mini EDTA-free and phosphoStop (Roche, Basel, Switzerland). After sonication three times with 4 s pulses for 20 s and incubation on ice for 30 min, a centrifugation at 16,000× *g* for 30 min (4 °C) was performed and the supernatant containing the extracted proteins was collected. A BCA assay was performed to determine protein concentration of the supernatant (Pierce BCA Protein Assay Kit, Thermo Fisher Scientific). Afterwards proteins underwent reduction with dithiothreitol (F.c 10 mM DTT) at 56 °C for 30 min and carbamidomethylation with iodoacetamide (F.c. 30 mM IAA) for 30 min in the dark. Next, 100 µg of proteins were subjected to the filter-aided sample preparation (FASP) using 30 kDa weight cut-off (MWCO) membrane filter (Pall Corporation, Nanosep, Post Washington, New York, NY, USA)) and 100 µL of 8 M Urea in Tris-HCl buffer (pH 8.5). Centrifugation for three times at 13,500× *g* at RT was applied to wash the proteins in the filter. Afterwards, three washing steps with 50 mM aminobicarbonate (NH_4_HCO_3_) buffer (pH 7.8) were performed in the same manner as with urea buffer. Lastly, proteins were subjected into 100 µL tryptic digestion buffer of 4 µg trypsin (Promega, Trypsin Gold, Mass Spectrometry Grade, Madison, WI, USA) in 2 mM CaCl_2_, 0.2 GuHCl and 50 mM ABC (pH 7.8) for 14 h at 37 °C. Next day, the tryptic peptides were collected using centrifugation for 13,500× *g* for 15 min, together with 50 µL of 50 mM ABC buffer (pH 7.8) and then with 50 µL of ultra-pure H_2_O. Subsequently, an acidification step with 10 % TFA (*v*/*v*) to completely inactivate trypsin was introduced to the tryptic mixture and peptides were stored at 4 °C or −20 °C for short-term usage. The digestion efficiency was controlled by reversed-phase chromatography on an Ultimate 3000 (Thermo Fisher Sccientific) equipped with a PepSwift Monolithic Rapis Seperation Liquid Chromatography(RSLC), 200 µm × 5 cm, column.

### 4.7. Nano LC-ddMS/MS Analysis and Label-free Protein Quantitation

LC-ddMS/MS analysis was performed on an Ultimate U3000 nano RSLC system coupled to an Orbitrap Fusion™ Lumos™ Tribrid™ Mass Spectrometer (Thermo Fisher Scientific). First, tryptic peptides were pre-concentrated on an Acclaim PepMap 100 µm × 2 cm, C18, 5 µm, 100 Å trap column using 0.1% TFA (*v*/*v*) for 10 min, at a flow rate of 20 µL/min. Next, peptide separation on an Acclaim PepMap RSLC 75 µm × 50 cm, C18, 2 µm, 100 Å main column using a linear gradient from 97% solvent A (0.1% formic acid) and 3% solvent B (84% acetonitrile in 0.1% formic acid) to 35% solvent B over 120 min at a flow rate of 250 nL/min. The ddMS/MS data acquisition was set to obtain MS scan in the orbitrap from 300 to 1500 *m*/*z* at a resolution of 120,000, polysiloxane ion at 445.12002 *m*/*z* was used as lock mass. The most intense ions were then subjected to high collision induced dissociation (HCD), MS/MS scans were detected in the linear ion trap. The ion accumulation time was set to 300 ms (MS) and 50 ms (MS/MS). The automatic gain control (AGC) was set to 2 × 106 for MS survey scans and 2 × 104 for MS/MS scans. 

Data analysis of the acquired label-free quantitation started with importing MS raw files for three biological replicates of *Sh3tc2^ΔEx1/ΔEx1^* mice sciatic nerves and three corresponding controls into the Progenesis Qi LC-MS software from Nonlinear Dynamics (Newcastle upon Tyne, UK). Progenesis Qi selected one of the LC-MS files as reference to align all the MS raw data. Peak-picking step within 0–120 min retention time, mass to charge ratios of 300–1500 and charge states of +2, +3, or +4 were selected for peptide statistics. MS/MS spectra exported directly into a mgf file from Progenesis Qi were subsequently searched against a concatenated target/decoy database of mouse proteome (downloaded from Uniprot in July 2015 containing 16,716 target sequences). SearchGUI 3.2.20 and Mascot 2.4.0 (Matrix Science) were chosen as search engines with usage of X!Tandem Jackhammer (2015.12.15.2), MS-GF+ Beta (v10282) (19 Decenmber 2014) algorithms. The search criteria in each engine were set as: a maximum of two missed cleavages for trypsin, carbamidomethylation of cysteine and oxidation of methionine as fixed and variable modifications, respectively, 10 ppm and 0.5 Da for MS and MS/MS tolerances, respectively. PeptideShaker 22 1.16.15 was used for interpretation of identified peptides and proteins from all search engines (SearchGUI and Mascot) with a false discovery rate (FDR) of 1% on the protein level. The database search results were then imported back into Progenesis Qi. Afterwards, proteins that were quantified with unique peptides were exported and the normalized abundances’ average of each protein (obtained from Progenesis) from the triplicate analyses was calculated to determine the ratios between the CMT4C and control nerve samples.

### 4.8. Statistical Analysis

All statistical analysis was performed using GraphPad Prism V7, GraphPad, San Diego, CA, USA). Data was tested for normality using the D’Agostino-Pearson omnibus normality test and outliers were identified using the ROUT (Q = 1%) method. Normally distributed data were analysed using students *t*-test, while non-parametric data was analysed using the Mann-Whitney test, with *p* < 0.5 being considered significant. The removal of outliers did not affect the statistical tests in terms of significance levels and thus all data points are included in the graphs unless otherwise stated. All data and graphs show mean ± standard error of mean (SEM), with control animal values being given first followed by *Sh3tc2^ΔEx1/ΔEx1^* animals. * *p* < 0.05, ** *p* < 0.01, *** *p* < 0.001, **** *p* < 0.0001.

## 5. Conclusions

In conclusion, a murine model for the human disease CMT4C, the *Sh3tc2*^Δ*Ex1*/Δ*Ex1*^ mouse, shows NMJ alterations on a structural and transcriptional level, supported by proteome findings in the sciatic nerve, that could contribute to the pathomechanisms of the disease. The increase in branching at the NMJ of *Sh3tc2*^Δ*Ex1*/Δ*Ex1*^ mice may be an attempt at adapting to the pathology and is probably mediated by an increase in neurotrophic factors produced by muscle and proteomic changes in the sciatic nerve. If NMJ vulnerability is demonstrated in patients then it extends the list of neurodegenerative conditions in which the NMJ is a site of pathology and may offer a potential therapeutic target.

## Figures and Tables

**Table 1:** List of relevant proteins and their subcellular localizations and functions identified with altered abundances in sciatic nerves from eight-month old *Sh3tc2*^Δ*Ex1*/Δ*Ex1*^ mice. All listed proteins have been quantified based on at least one unique peptide. ECM: extra-cellular matrix.

Accession #	Protein Name	Unique Peptides	Fold of Regulation (Log2)	*p*-ANOVA	Localization	Function
Q8BGY2	Eukaryotic translation initiation factor 5A-2 (IF5A2_MOUSE)	2	−2.45	0.001	Nucleus/ ER	Mediates effects of polyamines on neuronal process extension and survival
Q62264	Thyroid hormone-inducible hepatic protein (THRSP_MOUSE)	1	−2.36	0.048	Nucleus	Mediates biosynthesis of triglycerides with medium-length fatty acid chains and modulates transcription factor activity of THRB
Q8K0E8	Fibrinogen beta chain (FIBB_MOUSE)	24	2.06	0.006	ECM	Polymerizes to form an insoluble fibrin matrix
P03995	Glial fibrillary acidic protein (GFAP_MOUSE)	5	2.08	0.009	Cytoplasm	Class-III intermediate filament; cell-specific marker that, during the development of the central nervous system, distinguishes astrocytes from other glial cells
Q8BK62	Olfactomedin-like protein 3 (OLFL3_MOUSE)	4	2.14	<0.0005	ECM	Critical for early development and functional organization of the nervous system as well as hematopoiesis
Q60847	Collagen alpha-1(XII) chain (COCA1_MOUSE)	51	2.16	<0.0005	ECM	In general, collagens have been described to be important for the structural and functional integrity of the neuromuscular junction
P21981	Protein-glutamine gamma-glutamyltransferase 2 (TGM2_MOUSE)	12	2.17	<0.0005	Multiple	Catalyzes the cross-linking of proteins and the conjugation of polyamines to proteins
P14106	Complement C1q subcomponent subunit B (C1QB_MOUSE)	1	2.21	0.047	ECM	First component of the serum complement system
P08121	Collagen alpha-1(III) chain (CO3A1_MOUSE)	3	2.42	0.042	ECM	In general, collagens have been described to be important for the structural and functional integrity of the neuromuscular junction
Q8VCM7	Fibrinogen gamma chain (FIBG_MOUSE)	20	2.47	0.004	ECM	Polymerizes to form an insoluble fibrin matrix
Q61001	Laminin subunit alpha-5 (LAMA5_MOUSE)	24	2.50	0.020	ECM	Mediate the attachment, migration and organization of cells into tissues during embryonic development by interacting with other extracellular matrix components
P55002	Microfibrillar-associated protein 2 (MFAP2_MOUSE)	3	2.78	0.050	ECM	Fibrinogen binding; component of the elastin-associated microfibrils
P51910	Apolipoprotein D (APOD_MOUSE)	9	2.92	<0.0005	ECM	Glia-derived apolipoprotein required for peripheral nerve functional integrity and a timely response to injury
Q8CC35	Synaptopodin (SYNPO_MOUSE)	1	3.02	0.005	Cytoskeleton	modulating actin-based shape and motility of dendritic spines; involved in synaptic plasticity
P11087	Collagen alpha-1(I) chain (CO1A1_MOUSE)	8	3.08	0.036	ECM	In general, collagens have been described to be important for the structural and functional integrity of the neuromuscular junction
P22935	Cellular retinoic acid-binding protein 2 (RABP2_MOUSE	2	3.14	<0.0005	Nucleus/ ER	Participates in retinoic acid-modulated nerve regeneration
Q01149	Collagen alpha-2(I) chain (CO1A2_MOUSE)	7	3.32	0.033	ECM	In general, collagens have been described to be important for the structural and functional integrity of the neuromuscular junction
O08746	Matrilin-2 (MATN2_MOUSE)	11	3.40	0.003	ECM	Involved in matrix assembly and axon guidance
P08226	Apolipoprotein E (APOE_MOUSE)	18	3.52	<0.0005	ECM	Regulation of regeneration in the peripheral nervous system
Q2UY11	Collagen alpha-1(XXVIII) chain (COSA1_MOUSE	19	4.10	0.006	ECM	In general, collagens have been described to be important for the structural and functional integrity of the neuromuscular junction

## Supplementary Materials

Supplementary materials can be found at http://www.mdpi.com/1422-0067/19/12/4072/s1 and include a table of primer sequences and reaction conditions.

## Abbreviations

CMT	Charcot-Marie Tooth
SH3TC2	SH3 domain and tetratricopeptide repeats 2
Nrg1/ErbB	Neuregulin-1/ErbB
NMJ	Neuromuscular junction
tSC	Terminal Schwann cells
SEM	Standard Error of the Mean
AChRγ/α/ε	Acetylcholine Receptor gamma/alpha/epsilon
MuSK	Muscle Specific Kinase
NCAM	Neural cell adhesion molecule
NGF	Nerve growth factor
BDNF	Brain-derived neurotrophic factor
CNTF	Cilliary neurotrophic factor
GDNF	Glial cell derived neurotrophic factor
NTRK	Neurotrophic receptor tyrosine kinase
ECM	Extra-cellular matrix
MAPK	Mitogen activated protein kinases
GFAP	Glial fibrillary acidic protein
THRSP	Thyroid Hormone-Responsive
FIBB	Fibrinogen beta chain
FIBG	Fibrinogen gamma chain
MFAP2	Microfibrillar-associated protein 2
OLFL3	Olfactomedin-like protein 3
TGM2	Protein-glutamine gamma-glutamyltransferase 2
RABP2	Cellular retinoic acid-binding protein 2
C1QB	Complement C1q subcomponent subunit B
LAMA	Laminin subunit alpha
APOD/E	Apolipoprotein D/E
MATN2	Matrilin-2
SYNPO	Synaptopodin
pAB/mAB	Poly/mono-clonal antibody
IgG	Immunoglobulin g
PBS	Phosphate Buffered Saline
NTC	No template Control
RT	Room Temperature
CT	Cycle Threshold
BCA	bicinchoninic acid assay
MS	Mass spectrometry
