# Supplementary material for: Neuromuscular Junction Changes in a Mouse Model of Charcot-Marie-Tooth Disease Type 4C
Source: Int J Mol Sci. 2018 Dec 17;19(12):4072. doi: 10.3390/ijms19124072 (PMC6320960; doi:10.3390/ijms19124072)
Supplement: Supplementary file 1 [file ijms-19-04072-s001.pdf]

Supplementary Table 1. Showing primer sequences and reaction conditions of qPCR experiments.

Gene of Interest	Forward Sequence	Reverse Sequence	Annealing Temp (°C) and cDNA Dilution	NCBI Ref
TATA BBP	TGCCCAGCATCACTATTTCA	CCGTAAGGCATCATTGGACT	60; 1/5	NM_013684.3
ACHR α	TCCCTTCGATGAGCAGAACT	GGGCAGCAGGAGTAGAACAC	60; 1/5	NM_007389.5
ACHR ε	GCAGCTTTTACCGAGAATGG	CGTCAGTTTCTCCAGGACC	60; 1/5	NM_009603.1
ACHR γ	GACCAACCTCATCTCCCTGA	GAGAGCCACCTCGAAGACAC	60; 1/5	NM_009604.3
MUSK	TTCAGCGGGACTGAGAAACT	TGTCTTCCACGCTCAGAATG	61.5; 1/5	AY360453.1
BNDF	TAATGCAGCATGATGGGAAA	TCACAGTGAAAGCACCTTGC	60; 1/5	NM_001048139.1
NCAM	AAGGGGAAGGCACTGAATTT	TCTCCTGCCACTTGACACAG	60; 1/5	NM_001081445.1
GDNF	CAGCCCCTGCTTTCTATCTG	TATGTTCAGGGCTTCCAAGG	61; 1/5	NM_001301332.1
CNTF	CGCTGGAGTGAGATGACTGA	AGGCAGAAACTTGGAGCGTA	61; 1/5	NM_170786.2
NGF	GCAGTGAGGTGCATAGCGTA	CTGTGTCAAGGGAATGCTGA	60; 1/5	NM_001112698.2
NTRK2	CGGCACATAAATTTCACACG	GTGAGGTTAGGAGCAGCCAG	63.9; 1/5	NM_001282961.1
NTRK3	AAGTAACCGGCTCACCACAC	GATGCAGTAAAGGCTCTGGC	63.9; 1/5	NM_001164034.1
NGFR(P75)	CAACCAGACCGTGTGTGAAC	GAGAACACGAGTCCTGAGCC	63.9 ; 1/5	NM_033217.3
